# Predictors of Body Mass Index Change From 11 to 15 Years of Age: The 1993 Pelotas (Brazil) Birth Cohort Study

**DOI:** 10.1016/j.jadohealth.2012.08.012

**Published:** 2012-12

**Authors:** Maria Cecília F. Assunção, Ludmila C. Muniz, Samuel C. Dumith, Valerie L. Clark, Cora L.P. Araújo, Helen Gonçalves, Ana M.B. Menezes, Pedro C. Hallal

**Affiliations:** Postgraduate Program in Epidemiology, Federal University of Pelotas, Brazil

**Keywords:** Obesity, Adolescent, Cohort studies, Body mass index

## Abstract

**Purpose:**

We explored predictors of nutritional status change from 11 to 15 years of age by analyzing prospective data.

**Methods:**

We collected data at 11 and 15 years of age from individuals born in 1993 in Pelotas, Brazil. We assessed nutritional status using body mass index (BMI) for age in *z*-score according to the World Health Organization 2007 standards. Independent variables collected at 11 years of age were socioeconomic position, adolescent's perception of own weight, body dissatisfaction, and weight loss dieting.

**Results:**

Of the 4,032 adolescents whose nutritional status could be evaluated in the two follow-ups, 93% maintained their nutritional status classification from 11 to 15 years. A total of 102 (2.8%) became obese and 181 (4.5%) ceased to be obese in the 4-year period. The prevalence of obesity decreased from 11 to 15 years of age in both boys and girls. Low-income girls were more likely to become obese from 11 to 15 years of age compared with high-income ones. Among boys, those with high income were more likely to cease being obese compared those with low income. Those who perceived themselves to be obese, who wished to have a smaller silhouette, and who were on diets to lose weight were more likely to become obese or to achieve a normal BMI category at 15 years of age.

**Conclusions:**

BMI tracks strongly in early adolescence. This finding suggests that interventions to more effectively change nutritional status should be implemented in childhood and should consider emotional aspects as well as social and biological ones.

**Implications and Contribution**

Nutritional status tracks strongly in early adolescence, and low-income girls were more likely to become obese from 11 to 15 years of age compared with high-income ones. Among boys, those with high income were more likely to cease being obese compared with those with low income.

Adolescence has been identified as a stage of great vulnerability for the establishment of risk behaviors. Thus, messages and commercial information from the media, which are often negatively focused on obesity, may affect adolescents' perception of weight norms and may lead to weight misperception, especially self-perceived overweight and slimming behaviors [1,2]. Studies have shown that dieting and worries about weight may have a negative impact on health, including increased risk of obesity [3,4]. Likewise, studies have demonstrated that low socioeconomic status is associated with obesity in adolescents and adults [5–7].

In analyzing prospective data from a long-lasting Brazilian birth cohort, we observed that during early adolescence some individuals become obese whereas others cease being obese. The objective of this study was to determine whether behavioral and economic characteristics are related to new cases of obesity or new cases of normal weight.

## Methods

We considered all live births in 1993 (N = 5,265) in the city of Pelotas, Brazil, to be eligible for a birth cohort study, and included 5,249. There were 81 multiple births (1.5%). We visited subsamples of this cohort at 1, 3 and 6 months of life and at 1, 4, 6, and 9 years of age. Detailed information about the study is available elsewhere [8,9]. In 2004–2005 and 2008–2009, when the cohort participants were on average 11 and 15 years of age, respectively, we carried out follow-up visits aiming to find all participants of the cohort.

In both follow-ups, we obtained all information and measurements through interviews performed in households and conducted by trained interviewers, using the same methodology at each time point.

We performed adolescents' nutritional status assessment using body mass index (BMI) for age in *z*-scores. We calculated BMI based on two measures of weight and height and used the mean value in the analysis. We measured weight using a digital scale with precision of 100 g (SECA, Birmingham, UK). We measured height using an aluminum stadiometer with precision of 1 mm, made for the study. Interviewers underwent standardization testing [10] before beginning fieldwork and every 2 months afterward to determine repeatability and validity of measurements. We adopted the World Health Organization standards for children and adolescents aged 5–19 years [11]. Adolescents with BMI index for age >2 *z*-score were classified as obese. We created four situations based on the combination of the presence of obesity at 11 and 15 years of age, categorized as: non-obese at age 11 and 15 years; non-obese at age 11 years and obese at age 15 years; obese at age 11 years and non-obese at age 15 years; and obese at age 11 and 15 years.

We collected independent variables during the 11-year-old follow-up, except for the variable of sex, which we collected in the perinatal study. Other variables used were adolescent's perception of own weight, body dissatisfaction, and practice of weight loss dieting. We determined socioeconomic position (SEP) employing an index of assets constructed using principal component analysis, based on a list of 18 socioeconomic indicators. The sum of each individual variable was later transformed into a continuous variable and categorized into tertiles.

We determined body dissatisfaction by comparing desired body image with perceived body image. We assessed body image using the rating scale from Stunkard et al. [12]. This scale contains nine male and female figures ordered from the smallest to the largest silhouette. First, adolescents chose the figure they believed looked most like them; afterward, they elected the silhouette they wished to have. If the answer to both questions was the same silhouette, the adolescent was considered to be satisfied with his body image. Otherwise, the youth was considered to be dissatisfied with his body image when he wished to have a smaller or larger silhouette. Perception of own weight was categorized as very fat, fat, normal, thin, or very thin. We later grouped these options into three categories: fat, normal, and thin. We obtained the variable of weight loss dieting through adolescents' report of having gone on some type of diet or dietary restriction to lose weight in the past 12 months.

We analyzed data using Stata software, version 12.0 (Stata Corp., College Station, TX). The descriptive analysis included absolute numbers and percentages or means for all study variables. We performed multivariate analysis using Poisson regression with robust variance [13] and present the effects as prevalence ratios. We analyzed changes in BMI category in the 11- to 15-year period according to the independent variables adjusted for SEP. All analyses were stratified by sex. *p* < .05 was assumed for two-tailed tests.

The Ethics and Research Committee of the Medicine College at the Federal University of Pelotas approved the study. Parents or guardians signed a written informed consent form authorizing youths to participate in the study.

## Results

In 2004–2005, we located and interviewed 4,452 cohort members, which corresponds to a follow-up rate of 87.5%. In the 2008–2009 follow-up, we located 4,325 adolescents (85.7%). Table 1 describes nutritional status, adolescents' body image, and weight loss dieting at 11 years of age. About a third of the boys were already overweight or obese. This proportion was slightly lower among girls (28.1%); 21.7% of boys and 28.5% of girls reported feeling “fat.” Dissatisfaction with body image was present in more than half of adolescents; 31.5% of boys and 40.0% of girls would like to have had a smaller silhouette. The proportion of girls (10.1%) who reported having gone on a diet to lose weight in the 12 months preceding the interview was higher than that among boys (6.9%).

Table 2 displays mean BMI and BMI *z*-scores at 11 years of age among non-obese individuals according to the independent variables. Those who perceived themselves as fat at 11 years of age had a mean BMI of 20.1 kg/m^2^, whereas those who perceived themselves as thin had a mean BMI of 15.8 kg/m^2^. We saw similar findings in terms of body dissatisfaction. Those who reported having gone on a weight loss diet had a mean BMI of 20.6 kg/m^2^, compared with a mean value of 17.5 kg/m^2^ among those who had not.

The prevalence of obesity decreased among both girls and boys between the ages of 11 and 15 years. In boys, obesity decreased from 13.6% to 10.3% (*p* < .001) and in girls it fell from 7.8% to 7.2% (*p* < .001). In the whole sample, the prevalence of obesity fell from 10.7% to 8.7%.

**Figure 1:**
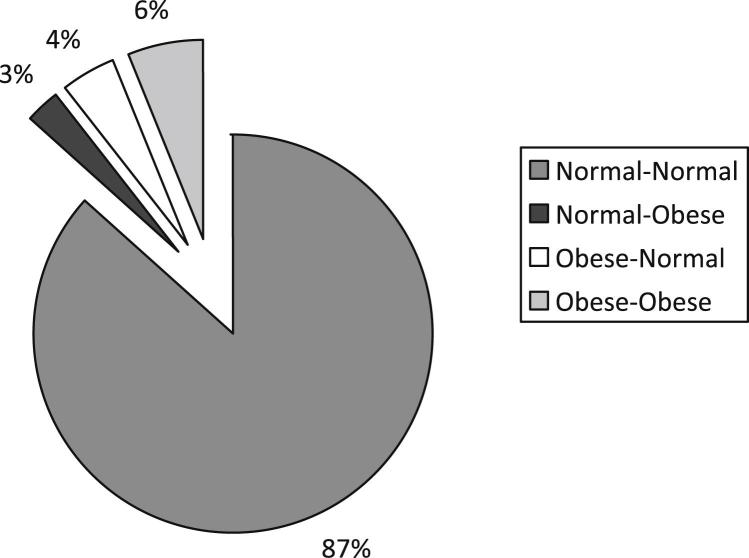
BMI from 11 to 15 years of age (n = 4,032).

Figure 1 shows that tracking of BMI was strong in the 4-year period. Of the 4,032 adolescents whose nutritional status could be evaluated in the two follow-ups, 3,749 (93%) maintained their nutritional status classification (87% normal–normal and 6% obese–obese). Among the others, 102 (3%) became obese in the period: 2.6% of boys (n = 45) and 3.0% of girls (n = 57) (*p* =.52). On the other hand, 181 adolescents (4%) ceased to be obese in this period: 5.0% of boys (n = 110) and 3.1% of girls (n = 71) (*p* = .002).

Table 3 shows the crude and adjusted analysis for the association between subjects who became obese or ceased to be obese and the independent variables in girls. In the crude analysis, girls belonging to the lowest tertile of SEP were more likely to become obese. Girls who classified themselves as fat and those who wished to have a smaller silhouette had a greater probability of becoming obese (prevalence ratio [PR] 3.2) or ceasing to be obese (PR 2.6) compared with those who classified themselves as normal or thin, or who wished to have a larger silhouette. Weight loss dieting was associate with the two outcomes (RP 3.1 for become obese and RP 5.4 for to ceasing to be obese). After adjustment for SEP, all associations were maintained in the same direction.

For the boys (Table 4 ) in the crude analysis, SEP was directly associated only with ceasing to be obese (PR 2.7 for adolescents of the highest tertile). In the crude and adjusted analysis, boys who perceived themselves as fat had increased probability of becoming obese or ceasing to be obese compared with those who perceived themselves to be at normal weight or thin. We found the same result for boys who wished to have a larger silhouette. As we saw among girls, weight loss dieting was associated with gaining or losing weight.

## Discussion

Through this longitudinal study, which follows up children since their birth in 1993 in the south of Brazil, we observed that obesity decreased in both boys and girls between 11 and 15 years of age. The prevalence of obesity in our cohort was in agreement with the National Adolescent School–based Health Survey conducted in 2009 [14].

Another interesting observation is the tracking of nutritional status that was very strong: <10% of teens had changed their BMI category in this period. This result is similar to a British study that found a tracking equivalent proportion of 82% from 11 to 16 years of age [15].

Because tracking of BMI is strong, it is unsurprising that 11-year-olds who perceived themselves as fat had a higher incidence of obesity from 11 to 15 years of age. This is explained by the actual higher mean BMI at baseline (11 years) than in those who perceived themselves as either normal or thin, as shown in Table 2. This finding indicates that when an adolescent gets on the path to obesity, it is difficult for him to change the eventual outcome. This is confirmed by our finding that only 4.5% of our cohort members were obese at 11 years and non-obese at 15 years. In the British study mentioned previously, only 1.4% of the adolescents ceased to be obese from 11 to 16 years of age [15].

Our study showed that lower SEP in early adolescence was associated with a higher risk of obesity at the age of 15 only among girls. This finding is in agreement with a longitudinal study conducted with Danish adolescents between the ages of 15 and 21 years, which showed that girls of lower parental SEP had a higher risk of developing overweight compared with girls of high parental SEP [6].

For boys, high SEP was a predictor of ceasing to be obese. We hypothesized that these boys may have greater access and adherence to healthy habits that promote weight loss. No studies have been found with a similar outcome.

Similar to other studies [1,2,16], we saw that weight misperception, body dissatisfaction, and slimming behaviors are highly prevalent among adolescents. Studies have mainly focused on obese subjects when documenting these behaviors [3,17]. Surprisingly, in our study, these feelings and behaviors were predictors of becoming obese or ceasing to be obese, even after adjustment for SEP. Our hypothesis is that other variables may be contributing to these findings in different ways: for example, depressive symptoms. Research into the association between depression and body size, an association that is likely to be bidirectional, has mainly focused on obesity. Nevertheless, a clinical perspective suggests that depression may be associated with underweight as well as overweight [18]. Further clarification should be given to these questions.

For boys and girls, we also found an association between weight loss dieting at the beginning of adolescence and becoming obese or ceasing to be obese. Studies that examined changes in BMI between adolescence and adulthood found a relationship between the practice of dieting to lose weight in adolescence and increased BMI in adulthood [19,20]. This is likely explained by poor adherence to weight loss dieting among adolescents. On the other hand, some teenagers can adhere to diets to lose weight and obtain satisfactory results. Moreover, other behaviors can interact with the diet and promote weight loss. We performed analyses comparing physical activity score (minutes per week in quartiles) and becoming or ceasing to be obese, but found no significant association (data not shown).

Some limitations of our research should be highlighted. We recognize that BMI is an imperfect indicator of adiposity because it does not distinguish lean mass from fat mass [21]. Others factors not explored in our report may also have a role, particularly pubertal status. We did not explore variable of puberty phenomena concerning ages of onset and ending, speed and magnitude of expression, and interrelations in this study. Such phenomena influence anthropometric and body composition changes. Because of this, to assess nutritional status change during adolescence, it is important to consider age and sexual maturity [22].

Another limitation is lack of knowledge about the nutritional status of the adolescents' parents, which is generally a strong predictor of overweight in early adulthood [23] and may explain a large amount of the variation and trajectories.

Given the strong tracking of BMI in early adolescence, strategies aimed at preventing obesity in populations should start in childhood. Also, interventions should consider emotional aspects as well as social and biological ones. There is evidence that interventions based on the transtheoretical model have the ability to approach multiple behaviors related to weight, including healthy eating, exercise, and management of emotional distress [24].

## Figures and Tables

**Table 1:** Description of study participants at age 11 years: 1993 Pelotas, Brazil, birth cohort study, 2004–2005 and 2008–009 follow-ups

Variable	Boys (N [%])	Girls (N [%])
Nutritional status	2,185	2,256
Underweight	30 (1.4)	54 (2.4)
Normal	1,429 (65.4)	1,567 (69.5)
Overweight	437 (20.0)	451 (19.9)
Obesity	289 (13.2)	184 (8.2)
Adolescent's perception of own weight	2,175	2,255
Fat	471 (21.7)	642 (28.5)
Normal	1,280 (58.9)	1,177 (52.2)
Thin	424 (19.4)	436 (19.3)
Body dissatisfaction	2,178	2,255
Wished to have smaller silhouette	685 (31.5)	902 (40.0)
Satisfied with body image	1,009 (46.3)	888 (39.4)
Wished to have larger silhouette	484 (22.2)	465 (20.6)
Weight loss dieting	2,187	2,260
No	2,035 (93.1)	2,032 (89.9)
Yes	152 (6.9)	228 (10.1)

**Table 2:** Mean body mass index at 11 years of age among non-obese adolescents according to perception of own weight, body dissatisfaction, and weight loss dieting: 1993 Pelotas, Brazil,) birth cohort study, 2004–2005 and 2008–2009 follow-ups (n = 4,441)

Variable	BMI (kg/m^2^) (mean [SD])	BMI *z*-scores (mean [SD])
Adolescent's perception of own weight		
Fat	20.1 (2.1)	1.6 (1.0)
Normal	17.6 (2.1)	.2 (1.0)
Thin	15.8 (1.5)	−.7 (.9)
Body dissatisfaction		
Wished to have smaller silhouette	19.5 (2.3)	1.3 (1.1)
Satisfied with body image	17.4 (2.0)	.1 (1.0)
Wished to have larger silhouette	16.0 (1.7)	−.6 (.9)
Weight loss dieting		
No	17.5 (2.3)	.2 (1.2)
Yes	20.6 (2.1)	1.8 (.9)

BMI = body mass index; SD = standard deviation.

**Table 3:** Crude and adjusted analysis of association between becoming obese or ceasing to be obese from 11 to 15 years of age, and independent variables in girls: 1993 Pelotas, Brazil, birth cohort study, 2004–2005 and 2008–2009 follow-ups

Variable	Became obese (n = 57)		Ceased to be obese (n = 71)	
	Crude analysis PR (95% CI)^a^	Adjusted analysis PR (95% CI)^a^	Crude analysis PR (95% CI)	Adjusted analysis PR (95% CI)^a^
Household assets index (tertiles)	*p* = .01^b^		*p* = .35^b^	
1° (lowest)	1.0		1.0	
2°	.4 (.2–.6)		1.7 (.9–3.0)	
3°	.4 (.2–.9)		1.4 (.8–2.5)	
Adolescent's perception of own weight	*p* < .001^c^	*p* < .001^c^	*p* < .001^c^	*p* < .001^c^
Fat	4.9 (2.8–8.0)	5.2 (2.9–9.1)	6.7 (3.8–11.8)	6.6 (3.8–11.7)
Normal	1.0	1.0	1.0	1.0
Thin	.1 (0–.1)	.1 (0–1.0)	.2 (0–1.4)	.9 (0–1.3)
Body dissatisfaction	*p* < .001^c^	*p* < .001^c^	*p* < .001^c^	*p* < .001^c^
Wished to have a smaller silhouette	2.9 (1.6–5.3)	2.9 (1.6–5.3)	12.8 (5.2–31.7)	12.9 (5.2–31.7)
Satisfied with body image	1.0	1.0	1.0	1.0
Wished to have a larger silhouette	.5 (.2–1.5)	.5 (.2–1.4)	0	.4 (0–3.2)
Weight loss dieting	*p* < .001^c^	*p* < .001^c^	*p* < .001^c^	*p* < .001^c^
No	1.0	1.0	1.0	1.0
Yes	3.1 (1.7–5.9)	3.3 (1.7–6.3)	5.4 (3.5–8.7)	5.4 (3.4–8.6)

CI = confidence interval; PR = prevalence ratio.

a Adjusted for Household Assets index at 11 years of age.

b Wald test for linear trend.

c Wald test for heterogeneity.

**Table 4:** Crude and adjusted analysis of association between becoming obese or ceasing to be obese from 11 to 15 years of age, and independent variables in boys: 1993 Pelotas, Brazil, birth cohort study, 2004–2005 and 2008–2009 follow-ups

Variable	Became obese (n = 45)		Ceased to be obese (n = 110)	
	Crude analysis PR (95% CI)	Adjusted analysis PR (95% CI)^a^	Crude analysis PR (95% CI)	Adjusted analysis PR (95% CI)^a^
Household assets index (tertiles)	*p* = .10^b^		*p* < .001^b^	
1° (lowest)	1.0		1.0	
2°	.9 (.5–1.8)		2.1 (1.2–3.7)	
3°	.9 (.5–1.8)		2.7 (1.6–4.6)	
Adolescent's perception of own weight	*p* < .001^c^	*p* < .001^c^	*p* < .001^c^	*p* < .001^c^
Fat	3.2 (1.8–5.9)	3.3 (1.8–6.1)	9.1 (5.9–14.0)	8.3 (5.4–12.9)
Normal	1.0	1.0	1.0	1.0
Thin	.2 (.1–.9)	.2 (0–.9)	0	0
Body dissatisfaction	*p* = .001^c^	*p* = .001^c^	*p* < .001^c^	*p* < .001^c^
Wished to have smaller silhouette	2.6 (1.4–4.7)	2.6 (1.4–4.8)	5.8 (3.7–9.2)	5.8 (3.6–9.5)
Satisfied with body image	1.0	1.0	1.0	1.0
Wished to have larger silhouette	.5 (.2–1.4)	.5 (.2–1.4)	.1 (0–.7)	.1 (0–.8)
Weight loss dieting	*p* = .001^c^	*p* = .002^c^	*p* < .001^c^	*p* < .001^c^
No	1.0	1.0	1.0	1.0
Yes	4.2 (1.7–10.2)	4.3 (.6–1.9)	4.6 (3.1–6.8)	3.8 (2.5–5.8)

Abbreviations as in Table 3.

a Adjusted for Household Assets index at 11 years of age.

b Wald test for linear trend.

c Wald test for heterogeneity.
